# Two Novel *Salmonella* Bivalent Vaccines Confer Dual Protection against Two *Salmonella* Serovars in Mice

**DOI:** 10.3389/fcimb.2017.00391

**Published:** 2017-09-04

**Authors:** Xinxin Zhao, Qinlong Dai, Renyong Jia, Dekang Zhu, Mafeng Liu, Mingshu Wang, Shun Chen, Kunfeng Sun, Qiao Yang, Ying Wu, Anchun Cheng

**Affiliations:** ^1^Research Center of Avian Diseases, College of Veterinary Medicine, Sichuan Agricultural University Chengdu, China; ^2^Key Laboratory of Animal Disease and Human Health of Sichuan Province Chengdu, China; ^3^Institute of Preventive Veterinary Medicine, Sichuan Agricultural University Chengdu, China

**Keywords:** *Salmonella* typhimurium, *Salmonella* newport, O-antigen, bivalent vaccines, protection

## Abstract

Non-typhoidal *Salmonella* includes thousands of serovars that are leading causes of foodborne diarrheal illness worldwide. In this study, we constructed three bivalent vaccines for preventing both *Salmonella* Typhimurium and *Salmonella* Newport infections by using the aspartate semialdehyde dehydrogenase (Asd)-based balanced-lethal vector-host system. The constructed Asd^+^ plasmid pCZ11 carrying a subset of the *Salmonella* Newport O-antigen gene cluster including the *wzx-wbaR-wbaL-wbaQ-wzy-wbaW-wbaZ* genes was introduced into three *Salmonella* Typhimurium mutants: SLT19 (Δ*asd*) with a smooth LPS phenotype, SLT20 (Δ*asd* Δ*rfbN*) with a rough LPS phenotype, and SLT22 (Δ*asd* Δ*rfbN* Δ*pagL::T araC* P_BAD_ *rfbN*) with a smooth LPS phenotype when grown with arabinose. Immunoblotting demonstrated that SLT19 harboring pCZ11 [termed SLT19 (pCZ11)] co-expressed the homologous and heterologous O-antigens; SLT20 (pCZ11) exclusively expressed the heterologous O-antigen; and when arabinose was available, SLT22 (pCZ11) expressed both types of O-antigens, while in the absence of arabinose, SLT22 (pCZ11) expressed only the heterologous O-antigen. Exclusive expression of the heterologous O-antigen in *Salmonella* Typhimurium decreased the swimming ability of the bacterium and its susceptibility to polymyxin B. Next, the *crp* gene was deleted from the three recombinant strains for attenuation purposes, generating the three bivalent vaccine strains SLT25 (pCZ11), SLT26 (pCZ11), and SLT27 (pCZ11), respectively. Groups of BALB/c mice (12 mice/group) were orally immunized with 10^9^ CFU of each vaccine strain twice at an interval of 4 weeks. Compared with a mock immunization, immunization with all three vaccine strains induced significant serum IgG responses against both *Salmonella* Typhimurium and *Salmonella* Newport LPS. The bacterial loads in the mouse tissues were significantly lower in the three vaccine-strain-immunized groups than in the mock group after either *Salmonella* Typhimurium or *Salmonella* Newport lethal challenge. All of the mice in the three vaccine-immunized groups survived the lethal *Salmonella* Typhimurium challenge. In contrast, SLT26 (pCZ11) and SLT27 (pCZ11) conferred full protection against lethal *Salmonella* Newport challenge, but SLT25 (pCZ11) provided only 50% heterologous protection. Thus, we developed two novel *Salmonella* bivalent vaccines, SLT26 (pCZ11) and SLT27 (pCZ11), suggesting that the delivery of a heterologous O-antigen in attenuated *Salmonella* strains is a prospective approach for developing *Salmonella* vaccines with broad serovar coverage.

## Introduction

Non-typhoidal *Salmonella* (NTS), a gram-negative and facultative intracellular bacterium, predominantly causes enteric diarrheal disease in a broad spectrum of animal hosts and humans, which represents a major public health concern, with an estimated 93.8 million cases worldwide and 155,000 deaths each year (Majowicz et al., 2010). NTS also infrequently causes serious dysentery and septicemia with substantial mortality, particularly in young infants, the elderly and immunocompromised individuals, such as HIV patients (Arshad et al., 2008; Parry et al., 2013). *Salmonella* infections have been linked to a variety of sources, particularly livestock and related products (e.g., poultry, eggs, beef, and dairy products) and produce (Braden, 2006; Scallan et al., 2011; Painter et al., 2013). NTS infections often result in an asymptomatic carrier state in adult domestic animals and the establishment of persistent infections, which have serious impacts on public health due to the risks of food poisoning via consumption of contaminated products. Furthermore, the prevalence of multidrug resistance among human and animal *Salmonella* isolates has increased over the past 2 decades (Threlfall, 2002; Varma et al., 2005; Karon et al., 2007). Thus, safe and effective NTS vaccines are urgently needed to prevent salmonellosis in both humans and domestic animals.

The global epidemiology of NTS diseases is complex, with diverse serovars in different regions worldwide, posing a substantial challenge for vaccine development. *Salmonella enterica* serovar Typhimurium (*Salmonella* Typhimurium) and *Salmonella* Enteritidis have been the most prevalent serovars in people and animals for many years; however, other serovars, such as *Salmonella* Newport and *Salmonella* Infantis have increasingly been detected in many countries in association with foodborne outbreaks (Bayer et al., 2014; Angelo et al., 2015). *Salmonella* Newport ranks among the top six serovars in the North American, European, and Latin American regions (Hendriksen et al., 2011); appears to be the most common serotype isolated from patients with diarrhea in some areas of China (Deng et al., 2012); and exhibits a high antimicrobial resistance rate in several countries (Espie et al., 2005; Centers for Disease Prevention, 2008 and Centers for Disease and Prevention, 2013). Additionally, immune cross-protection between serovar variants of *S. enterica* is limited due to the expression of different immunodominant O-antigens by these pathogens (Hormaeche et al., 1996). Thus, an ideal vaccine with broad serovar coverage should be developed to prevent diverse NTS serovar infections worldwide (Mahan et al., 2012); however, no licensed human NTS vaccines currently exist, and most human NTS vaccines in preclinical or clinical stages, such as the live vaccines CVD1921 and CVD1941 and the O-antigen-conjugated vaccine O:4,12-TT, are being explored for a single serovar, mainly Typhimurium or Enteritidis (MacLennan et al., 2014; Tennant and Levine, 2015). In addition, current commercial *Salmonella* vaccines used in animals exhibit efficacy but have limited cross-protection (Desin et al., 2013).

Oral live NTS vaccines can confer protection against salmonellosis by inducing both cell-mediated and humoral immune responses. To overcome the limitations of *Salmonella* vaccines, considerable efforts have been made to develop live attenuated vaccines with cross-protection. One strategy is the construction of live vaccines by mutating global regulators, leading to the overexpression of potential antigens that may be shared among heterologous serovars. The *Salmonella* Typhimurium double mutant Δ*cya* Δ*crp* induces cross-protection against heterologous serovars, including *Salmonella* Enteritidis and group C *Salmonella*, in chickens (Hassan and Curtiss, 1994). *Salmonella* Typhimurium DNA adenine methylase (Dam) mutants elicit protective immune responses to homologous and heterologous challenges in murine, avian and bovine animal models (Dueger et al., 2001; Heithoff et al., 2008; Mohler et al., 2008). However, the protection efficacies conferred by these vaccines against heterologous challenges are not complete and are not as good as their protective effects against the homologous strain (Dueger et al., 2001; Mahan et al., 2012; Heithoff et al., 2015). The other strategy is the introduction of mutations in genes required for lipopolysaccharide (LPS) or enterobacterial common antigen (ECA) biosynthesis, such as *rfaH* (Nagy et al., 2008), *rfbB* and *rffG* (Huang et al., 2016) and *wecA* (Bridge et al., 2015), resulting in the exposure of conserved outer membrane proteins (OMPs) to the host immune system. Likewise, the resulting vaccine strains, which exhibit a truncated LPS or ECA phenotype, can induce improved cross-immunity to heterologous bacteria but cannot provide full protection against heterologous serovars in most cases (Bridge et al., 2015; Huang et al., 2016). Thus, new approaches are required to develop multivalent *Salmonella* vaccines.

LPS O-antigens, composed of repeat-unit polysaccharides, are quite variable and determine the serogroup of *Salmonella*. Serovars in the same serogroup share the same dominant O-antigen epitope (Reeves et al., 2013). O-antigen-specific antibodies mediate strong protection against several homologous serovars of *S. enterica*, which is evidenced by O:4-specific IgG- or IgA-based adoptive transfer experiments (Colwell et al., 1984; Michetti et al., 1992; Forbes et al., 2012) and the potent efficacies displayed by O-antigen-conjugated vaccines, such as O:9-flagellin, O:4,12-TT, and O:4,5/O:9-CRM197 in animal models (Simon and Levine, 2012). Attenuated live *Salmonella* strains, which are ideal vaccine vectors with unparalleled merits, have been extensively used to deliver heterologous antigens from various pathogens and have been shown to induce protective responses against lethal challenges (Roland and Brenneman, 2013). These vaccine vectors are easy to handle and manipulate genetically and can be administered orally to both human and farm animals without the need for needles, inducing long-lasting systematic and mucosal immune responses against not only heterologous antigens but also the *Salmonella* carrier itself while causing few or no side effects when attenuated appropriately by deleting one or two key genes that are essential for virulence. In the past several decades, a variety of technologies, such as Asd (aspartate semialdehyde dehydrogenase)-based balanced-lethal vector-host systems and arabinose-dependent regulated delayed attenuation, have been exploited in *Salmonella* Typhimurium to enhance its efficacy as a vaccine vector (Wang et al., 2013). Asd is responsible for the synthesis of diaminopimelic acid (DAP), which is an essential component for the biosynthesis of cell walls in *Salmonella*; cell lysis will occur in its absence. The balanced-lethal host-vector system based on the essential bacterial gene for Asd has been developed to deliver heterologous antigens in a vaccine strain with the *asd* gene deleted from the chromosome using a recombinant plasmid carrying the wild-type *asd* gene to establish a complementation heterozygote. The absence of DAP in mammalian tissues drives *Salmonella* vaccine strains with an *asd* mutation to stably maintain Asd^+^ plasmids *in vivo*. This system possesses a safety advantage by avoiding drug-resistance gene markers in the development of recombinant vaccines. Additionally, a tightly regulated *araC* P_BAD_ activator-promoter has been frequently applied to replace upstream promoter sequences for O-antigen synthesis genes, making O-antigen expression dependent on arabinose provided during *in vitro* growth, which achieves regulated delayed attenuation. We hypothesized that immunization with a recombinant *Salmonella* Typhimurium vaccine strain stably expressing heterologous O-antigens from other serovars using the balanced-lethal vector-host system could provide protection against both homologous and heterologous *Salmonella* infections.

To address this issue, we attempted to construct recombinant *Salmonella* Typhimurium strains with heterologous expression of *Salmonella* Newport O-antigen. The genes specific for *Salmonella* O-antigen synthesis are generally present as a gene cluster in the chromosome, called the O-antigen gene cluster, which maps between the *galF* and *gnd* genes and is the major determinant of differences among the diverse O-antigen forms (Liu et al., 2014). The O-antigen gene cluster of *Salmonella* Newport includes 19 genes, among which the *wzx*-*wbaR*-*wbaL*-*wbaQ*-*wzy*-*wbaW*-*wbaZ* (*wzx*-*wbaZ*) gene sequences are distinct from or absent in the O-antigen gene cluster of *Salmonella* Typhimurium. Thus, we constructed a recombinant low-copy Asd^+^ plasmid, pCZ11, containing this subset of the *Salmonella* Newport O-antigen gene cluster (*wzx*-*wbaZ*) for heterologous O-antigen expression and introduced it into three *Salmonella* Typhimurium Δ*asd* mutant strains: SLT19 (ATCC14028 Δ*asd*), with a smooth LPS phenotype; SLT20 (ATCC14028 Δ*asd*Δ*rfbN*), with a rough LPS phenotype; and SLT22 (ATCC14028 Δ*asd* Δ*rfbN* Δ*pagL*::T *araC* P_BAD_ *rfbN*), with an arabinose-dependent LPS phenotype. This resulted in three recombinant strains expressing the heterologous *Salmonella* Newport O-antigen, SLT19 harboring the plasmid pCZ11 [herein termed SLT19 (pCZ11)], SLT20 (pCZ11), and SLT22 (pCZ11). The aim of the *asd* gene deletion was to ensure the stability of plasmid pCZ11 in the *Salmonella* Typhimurium Δ*asd* strains. *rfbN* was selected for gene deletion and gene regulation by the arabinose-dependent *araC* P_BAD_ promoter because it encodes a rhamnosyl transferase, which is required for the synthesis of *Salmonella* Typhimurium O-antigen but not *Salmonella* Newport O-antigen (Reeves et al., 2013). As the *rfbN* gene is located within the O-antigen gene cluster, the arabinose regulated gene cassette, T *araC* P_BAD_ *rfbN*, was inserted into the *pagL* site to achieve expression of *Salmonella* Typhimurium O-antigen in an arabinose-dependent manner. The deletion of *pagL* does not alter *Salmonella* virulence (Kong et al., 2010). Since O-antigens are associated with bacterial survival and virulence (Kong et al., 2011), we determined the effects of heterologous O-antigen expression on several *Salmonella* Typhimurium biological activities, including swimming, sensitivities to polymyxin B and sodium deoxycholate (DOC), colonization and virulence. The cAMP-receptor protein (CRP) is a global regulator of a number of genes involved in the uptake and utilization of carbon sources, flagellum synthesis, OMPs, etc., after binding to cAMP in *Escherichia coli* (*E. coli*) and *Salmonella* Typhimurium (Lawson et al., 2004). *Salmonella* with mutations in *crp* alone or in combination with other genes were shown to be greatly attenuated in virulence and could serve as effective vaccine candidates against salmonellosis (Chu et al., 2007). Thus, for attenuation and vaccine construction, we deleted the *crp* gene from the three recombinant strains and ultimately evaluated the immunogenicity and protective efficacy of the attenuated vaccine strains in BALB/c mice.

## Materials and methods

### Ethics statement

The treatment of animals in this study was in accordance with the Guide for the Care and Use of Laboratory Animals from the Ministry of Science and Technology of China. All animal protocols were approved by the Animal Ethics Committee at Sichuan Agricultural University and the Sichuan Administration Committee of Laboratory Animals under protocol number SYXK2014-187.

### Bacterial strains, plasmids, media, and growth conditions

The bacterial strains and plasmids used are listed in Table 1. The recombinant *Salmonella* Typhimurium strains were derived from the highly virulent ATCC14028 strain. The wild-type *Salmonella* Newport SLN06 strain was isolated from fecal samples from a duck farm in China and was virulent, with an oral LD_50_ of 2 × 10^7^ CFU in BALB/c mice (Table 2). All bacterial strains were grown in Luria-Bertani (LB) broth or on LB agar with or without 0.1% arabinose. When required, antibiotics and supplements were added at the following concentrations: ampicillin, 100 μg/ml; chloramphenicol, 25 μg/ml; and diaminopimelic acid (DAP), 50 μg/ml. DAP is essential for the growth of the *Salmonella* Typhimurium Δ*asd* mutant. LB agar without NaCl and containing 10% sucrose was used for *sacB* gene-based counterselection in the allelic exchange experiments. The transformation of *E. coli* and *Salmonella* Typhimurium was performed via electroporation. Transformants were selected on LB agar plates containing the appropriate antibiotics.

**Table 1:** Bacterial strains and plasmids used in this study.

**Strains or plasmids**	**Description**	**Source**
**PLASMIDS**		
pKS011	PLtetO-1 pSC101 origin, Cm^r^	Sekar et al., 2016
pTRC-LIC	Ptrc pBR322 origin, Amp^r^	Addgene
BBa_J72113-BBa_J72152	*araC* P_BAD_p15a origin, Cm^r^ Amp^r^	Kittleson et al., 2012
pRE112	*sacB* mobRP4 R6K ori Cm^+^	Edwards et al., 1998
*E. coli* SM10*λpir*	*thi thr*-1 *leu*6 *pro*A2 *his*-4 *arg* E2 *lac*Y1 *galK*2, *ara*14*xy*l5 *supE*44*,λpir*	Rubires et al., 1997
PCZb0	Asd^+^ Ptrc *sacB* pSC101 origin, Amp^r^	This work
pCZb1	Asd^+^ Ptrc pSC101 origin, Amp^r^	This work
pCZ11	Asd^+^ vector expressing *wzx*-*wbaZ* gene of *Salmonella* Newport O-antigen gene cluster	This work
pCZ12	pRE112-Δ*asd*	This work
pCZ13	pRE112-Δ*rfbN*	This work
pCZ14	pRE112-Δ*pagL*	This work
pCZ15	pCZ14-T *araC* P_BAD_ *rfbN*	This work
pCZ16	pRE112-Δ*crp*	This work
**STRAINS**		
ATCC14028	Wild-type *Salmonella* Typhimurium	ATCC
ATCC27869	Wild-type *Salmonella* Newport	ATCC
SLN06	Wild-type *Salmonella* Newport	Lab collection
SLT19	ATCC14028 Δ*asd rfbN*	This work
SLT20	SLT19Δ*rfbN*	This work
SLT21	SLT19 Δ*rfbN*Δ*pagL*	This work
SLT22	SLT19 Δ*rfbN*Δ*pagL*::T *araC* P_*BAD*_ *rfbN*	This work
SLT25	SLT19 Δ*crp*	This work
SLT26	SLT20 Δ*crp*	This work
SLT27	SLT22 Δ*crp*	This work

**Table 2:** Virulence of the wild-type *Salmonella* strains and the recombinant vaccines.

**Strains**	**LD_50_ (CFU)**
*Salmonella* Typhimurium ATCC14028	5 × 10^5^
*Salmonella* Newport SLN06	2 × 10^7^
SLT19 (pCZb1)	5 × 10^5^
SLT19 (pCZ11)	1.4 × 10^6^
SLT20 (pCZb1)	>8 × 10^9^
SLT20 (pCZ11)	2.7 × 10^6^
SLT22 (pCZb1)	>6 × 10^9^
SLT22 (pCZ11)	8 × 10^5^
SLT25(pCZb1)	>5 × 10^9^
SLT25(pCZ11)	>5 × 10^9^
SLT26(pCZb1)	>2 × 10^9^
SLT26(pCZ11)	>2 × 10^9^
SLT27(pCZb1)	>6 × 10^9^
SLT27(pCZ11)	>6 × 10^9^

### Plasmid construction

The primers used in this study are listed in Table S1. The Asd^+^ plasmid pCZb1 was constructed according to the plasmid pYA3337 (Baek et al., 2009). To construct the plasmid pCZb0, the primer pair pSC101-2F and pSC101ori-R was used to amplify a DNA fragment containing the lambda t0 terminator and pSC101 origin from the plasmid pKS011 (Sekar et al., 2016), and the primers Pasd-F and pSC101-2R were used to amplify the *asd* gene cassette from the ATCC14028 genome. The two fragments were then joined by PCR using the primers pSC101-2F and pSC101-2R. The product was then assembled by incubating at 50°C for 1 h with a DNA fragment containing the TIT2 terminator, Ptrc promoter, *sacB* gene cassette and ampicillin-resistance cassette cloned from the plasmid pTRC-LIC with the primers pSC101-1F and pSC101-1R to form the plasmid pCZb0 using a Gibson Assembly Kit (NEB, Beverley, MA, USA). The pKS011 and pTRC-LIC plasmids were gifts from Keith Tyo (Addgene plasmid #65464) and Cheryl Arrowsmith (Addgene plasmid #62343), respectively. Next, to remove the *sacB* gene cassette from plasmid pCZb0, the DNA fragment containing the TIT2 terminator and ampicillin-resistance cassette cloned from pCZb0 with the primers TIT2-F and pSC101-1R was assembled with the DNA fragment containing the lambda t0 terminator, pSC101 origin, *asd* gene cassette and Ptrc promoter cloned from pCZb0 by the primers pSC101-2F and Ptrc-R using a Gibson Assembly Kit (NEB), forming the plasmid pCZb1. Furthermore, to express the *Salmonella* Newport O-antigen in *Salmonella* Typhimurium, the recombinant plasmid pCZ11 was constructed based on pCZb1. In brief, the primers C2-O-antigen-F and C2-O-antigen-R were used to amplify the gene cluster *wzx*-*wbaR*-*wbaL*-*wbaQ*-*wzy*-*wbaW*-*wbaZ* (7,547 bp) from the *Salmonella* Newport ATCC27869 genome, and the primers pCZb1-C2F and pCZb1-C2R were used to amplify the entire coding sequence of plasmid pCZb1. Finally, the two PCR products were joined using a Gibson Assembly Kit (NEB), generating the recombinant plasmid pCZ11. The plasmid construction is depicted in Figure S1.

### Construction of bacterial mutant strains and recombinant strains

*Salmonella* Typhimurium mutant strains were constructed by allelic exchange using the suicide vector pRE112, which carries a chloramphenicol resistance gene and the sucrose-sensitivity gene *sacB* (Edwards et al., 1998), a gift from Dieter Schifferli (Addgene plasmid #43828), as previously described (Zhou et al., 2014). To delete the *rfbN* gene, the primer pairs D*rfbN*-1F/D*rfbN*-1R and D*rfbN*-2F/D*rfbN*-2R were used to amplify the regions ~400 bp upstream and downstream of the ATCC14028 genome, respectively. The two fragments were then joined by PCR using the primers D*rfbN*-1F and D*rfbN*-2R. The resulting PCR product was digested with *Kpn*I and *Xma*I and ligated into plasmid pRE112 that had been digested with the same enzymes to generate plasmid pCZ13, which carries a deletion of the entire *rfbN* gene sequence. pCZ13 was subsequently introduced into *Salmonella* Typhimurium ATCC14028 by *E. coli* SM10λ*pir* (Rubires et al., 1997) via conjugation. Recipient cells were cultured on LB agar supplemented with chloramphenicol to select for a trans-conjugant strain of ATCC14028 (pCZ13) that contained pCZ13 integrated into the *Salmonella* genome after a single crossover. Next, a colony of ATCC14028 (pCZ13) was grown in LB to allow for a second crossover. After overnight growth, the ATCC14028 (pCZ13) culture was plated on LB agar containing 10% (w/v) sucrose for the counterselection of colonies that lost the pRE112 vector carrying the counterselectable marker, *sacB*. Expression of *sacB* in *Salmonella* is lethal in the presence of sucrose (Edwards et al., 1998). The colonies that grew on the LB agar containing sucrose were then tested for chloramphenicol sensitivity to ensure loss of the plasmid. The *rfbN* mutants were finally confirmed by PCR using the primers D*rfbN*-1F and D*rfbN*-2R. The same method was used to delete the *asd, pagL*, and *crp* genes. To construct the arabinose-regulated mutants, the primers *rfbN*-F and *rfbN*-R were used to amplify the *rfbN* gene from the ATCC14028 genome, and the primers T *araC* P_BAD_-F and T *araC* P_BAD_-R were used to amplify the T *araC* P_BAD_ DNA fragment from the plasmid BBa_J72113-BBa_J72152 (Kittleson et al., 2012), a gift from Christopher Anderson (Addgene plasmid #40784). The two fragments were then joined by PCR using primers T *araC* P_*BAD*_-F and *rfbN*-R. The product was inserted into the *Not*I and *Sbf* I double-digested pCZ14 (pRE112-Δ*pagL*) plasmid, generating the plasmid pCZ15, which was introduced into the SLT21 strain (ATCC14028 Δ*asd* Δ*rfbN* Δ*pagL*) to insert T*araC*P_BAD_ *rfbN* into the *pagL* gene site, generating the SLT22 strain. In addition, for heterologous O-antigen expression, pCZ11 and the control plasmid pCZb1 were individually transformed into *Salmonella* Typhimurium SLT19 (Δ*asd*), SLT20 (Δ*asd* Δ*rfbN*) and SLT22 (Δ*asd* Δ*rfbN* Δ*pagL*::T *araC* P_BAD_ *rfbN*), generating three recombinant strains, SLT19 (pCZ11), SLT20 (pCZ11), and SLT22 (pCZ11), and three control strains, SLT19 (pCZb1), SLT20 (pCZb1), and SLT22 (pCZb1). The Asd^+^ plasmid and *Salmonella* Typhimurium Δ*asd mutant* constituted the balanced-lethal host-vector system. Loss of the plasmid is lethal for the Δ*asd* mutant.

### Sodium dodecyl sulfate-polyacrylamide gel electrophoresis (SDS-PAGE), silver staining and immunoblot analysis

To confirm the expression of *Salmonella* Typhimurium and *Salmonella* Newport O-antigens, silver staining and Western immunoblot analyses were performed as previously described (Xu et al., 2002; Digiandomenico et al., 2004). Whole-cell lysates of *Salmonella* organisms were separated on 12% (w/v) acrylamide gels using a Tricine-SDS buffer system (Bio-Rad Laboratories, California, USA). The gels were then silver-stained or analyzed by Western blotting with *Salmonella* O:4-specific or O:8-specific antiserum diluted to 1:200 (Tianjin Biochip Corporation, Tianjin, China).

### Swimming and sensitivity assays

*Salmonella* Typhimurium strains were grown to an OD_600_ of 0.8–0.9 in LB broth with or without 0.1% arabinose, harvested, and washed in phosphate-buffered saline (PBS). For the swimming assay, 6 μl of bacterial suspension at a concentration of 1 × 10^8^ CFU/ml was spotted onto the middle of an LB plate solidified with 0.25% agar and supplemented with or without arabinose. The colony diameters were measured after incubation at 37°C for 6 h. For the sensitivity assays, 100 μl of a 100-fold-diluted bacterial culture (~1 × 10^6^–5 × 10^6^ CFU) was inoculated with or without polymyxin B at a final concentration of 0.12 μg/ml or DOC at a final concentration of 4 mg/ml for 1 h at 37°C. The bacteria were diluted to the appropriate concentration and plated onto LB plates. The survival rate was calculated as the CFU per ml of the polymyxin B- or DOC-treated group divided by the CFU per ml of the non-treated group. Each experiment was repeated three times.

### Determination of bacterial colonization and virulence (50% lethal dose, LD_50_) in mice

Six-week-old female BALB/c mice were obtained from Dashuo Experimental Animal, Ltd. (Chengdu, China) and acclimated for 7 days after arrival before inoculation. The colonization ability and LD_50_ of *Salmonella* Typhimurium strains were determined as previously described (Kong et al., 2011). In brief, BALB/c mice were inoculated orally with 20 μl of buffered saline with gelatin (BSG) containing 1 × 10^9^ CFU of bacteria. For arabinose-regulated strains, 0.1% arabinose was added to the culture medium before inoculation. Six days after oral inoculation, four animals per group were euthanized by CO_2_ asphyxiation, and tissues including the Peyer's patches (PPs), spleen and liver were collected. The weight of each sample was measured, and the samples were homogenized in a total volume of 1 ml of BSG. Then, appropriate dilutions were plated onto MacConkey agar or LB agar to determine the number of viable bacteria. The colonization value was calculated as CFU per gram of tissue (CFU/g). To measure the LD_50_ of *Salmonella* Typhimurium strains, stepwise increasing doses of *Salmonella* Typhimurium strains in 20-μl bacterial suspensions were orally inoculated into groups of BALB/c mice (6 mice per group). For arabinose-regulated strains, 0.1% arabinose was added to the culture medium before inoculation. The mice were observed for 1 month after infection, and the mortality rates were recorded to calculate the LD_50_ of the strains using the method of Reed and Muench. To minimize suffering, all animals that displayed extreme signs of moribundity, including shallow breathing, shaking, unresponsiveness to touch, and an inability to move and obtain food and water, were immediately euthanized by CO_2_. The carcasses of dead animals were sterilized, enclosed and delivered to the laboratory animal center of Sichuan Agricultural University for bio-safety disposal.

### Plasmid stability of recombinant *Salmonella* vaccine strains

The plasmid stability *in vitro* was determined as previously described (Xin et al., 2012). An overnight culture of *Salmonella* Typhimurium strains carrying Asd^+^ vector pCZ11 was diluted 1:100 into fresh LB medium containing DAP (non-selective media) for 12 h of incubation with rotation at 37°C (T0). Then, serial dilutions of the culture were plated on LB plates containing DAP. The process described above was repeated every 12 h five times, and the culture from the final passage was considered T5 (~50 generations). Before each passage, a sample from the culture was diluted and plated onto LB plates containing DAP. Then, 100 single colonies from overnight growth were selected and streaked on LB agar plates without DAP (selective media) and on LB agar plates containing DAP (non-selective media). Plasmid stability was determined as the percentage of the 100 selected colonies that grew on selective media after each of the five passages.

The plasmid stability *in vivo* was determined as previously described (Gahan et al., 2007). Groups of BALB/c mice (12 mice/group) were inoculated orally with ~1 × 10^9^ CFU of *Salmonella* Typhimurium strains carrying Asd^+^ vector pCZ11. Four mice in each group were euthanized by CO_2_ asphyxiation, and PPs were collected at 3, 6, and 9 days post-infection. The PPs were weighed and homogenized in a total volume of 1 ml of BSG. Then, to determine the number of viable bacteria, appropriate dilutions were plated onto both LB agar plates without DAP (selective media) and LB plates containing DAP (non-selective media).

### Immunization and protection in mice

The *Salmonella* Typhimurium vaccine strains SLT25 (pCZb1), SLT25 (pCZ11), SLT26 (pCZb1), SLT26 (pCZ11), SLT27 (pCZb1), and SLT27 (pCZ11) were grown statically overnight in LB broth at 37°C. For arabinose-regulated strains, 0.1% arabinose was added to the culture medium. The following day, 1 ml of the overnight culture was inoculated into 100 ml of LB broth (arabinose was added to the arabinose-regulated strain cultures) and grown with shaking at 37°C to an OD_600_ of 0.8–0.9. The cells were harvested by centrifugation at 4,500 rpm for 10 min and resuspended in 1 ml of BSG. Groups of mice (16 mice per group) were orally inoculated with 20 μl of BSG containing 1 × 10^9^ CFU of each strain or with BSG without bacteria and were boosted with the same dose of each strain 4 weeks later. Serum was collected from 8 mice in each group via tail-vein bleeds or retroorbital venous plexus at 3 weeks after each immunization. One month after the second immunization, the mice in each group were challenged by oral inoculation with at least 100 × LD_50_ of the *Salmonella* Typhimurium virulent strain ATCC14028 or *Salmonella* Newport virulent strain SLN06. PP, spleen, and liver tissues were collected from 4 mice in each group 6 days post-challenge, and the bacterial count in each tissue was measured. The mortality of the remaining 12 mice was recorded daily for 21 days.

### Antigen preparation and quantitative enzyme-linked immunosorbent assay (ELISA)

*Salmonella* Typhimurium LPS was purchased from Sigma (St. Louis, MO, USA), and *Salmonella* Newport LPS was extracted and purified as previously described (Rezania et al., 2011). Serum IgG against *Salmonella* Typhimurium LPS or *Salmonella* Newport LPS was measured using a quantitative ELISA as previously described (Huang et al., 2016). In brief, a 96-well ELISA microplate was coated with 100 ng/well LPS or 100 ng/well goat anti-mouse Ig(H+L) (BD, San Diego, CA) in PBS and then blocked with 2% BSA (BD) for 2 h at room temperature after an overnight incubation at 4°C. The serum samples were diluted to 1:100 in PBS containing 2% BSA, and 100 μl of this solution was added to the LPS-coated wells in triplicate. Meanwhile, serial 2-fold dilutions of mouse IgG (BD) in 100 μl, starting from 0.5 mg/ml, were added to the goat anti-mouse Ig(H+L)-coated wells to generate a standard curve. After 1 h of incubation at 37°C, 100 μl of biotinylated goat anti-mouse IgG (Southern Biotech, Birmingham, AL, USA), biotinylated goat anti-mouse IgG1 (Southern Biotech) or biotinylated goat anti-mouse IgG2a (Southern Biotech) and 100 μl of a streptavidin-alkaline phosphatase conjugate (Southern Biotech) were added to each well sequentially followed by p-nitrophenyl phosphate substrate in diethanolamine buffer. Finally, the plate was read at 405 nm using a microplate reader (Bio-Rad Laboratories). The standard curve was drawn using Curve Expert (Hyams DG, Starkville, MS, USA), and the concentration of serum antibodies was calculated according to the standard curve.

### Serum bactericidal assay

The serum collected from mice in each immunized group at week 3 post-second immunization was pooled for serum bactericidal assays as described previously (Rondini et al., 2015). Commercially available *Salmonella* O:4-specific and O:8-specific antisera (Tianjin Biochip Corporation) were used as positive controls. All serum samples were heated at 56°C for 30 min to inactivate endogenous complement. *Salmonella* Typhimurium ATCC14028 or *Salmonella* Newport SLN06 were grown in LB medium to log phase and were diluted in SBA buffer (50 mM phosphate, 0.041% MgCl_2_·6H_2_O, 33 mg/ml CaCl_2_, and 0.5% BSA) to ~3 × 10^3^ CFU/ml. The bacteria were incubated with 25% heat-inactivated serum supplemented with or without active guinea pig complement (Sigma) for 1.5 h. The relative survival was calculated as the percent CFU counted in each pooled serum sample with active complement compared to the CFU of the same serum with no complement. Each sample and control was tested in triplicate.

### Statistical analysis

The data are shown as the means ± SD. One-way ANOVA followed by Tukey's multiple-comparison test was used to evaluate statistical significance. A probability value of *P* < 0.05 was considered statistically significant. The correlation between serum IgG concentrations post-immunization and bacterial loads post-challenge was analyzed using Spearman's rank correlation test in GraphPad Prism (GraphPad Software, California, USA). All *in vitro* experiments were repeated at least three times, and the *in vivo* experiments were repeated twice.

## Results

### Characterization of the constructed recombinant *Salmonella* typhimurium strains

To express the heterologous *Salmonella* Newport O-antigen in *Salmonella* Typhimurium, the control Asd^+^ plasmid pCZb1 and the recombinant Asd^+^ plasmid pCZ11 carrying the O-antigen gene cluster (*wzx*-*wbaZ*) of *Salmonella* Newport ATCC27869 were constructed first. Maps of the two plasmids are shown in Figure S1. To maintain the Asd^+^ plasmid in *Salmonella* Typhimurium, an ATCC14028 Δ*asd* mutant termed SLT19 was constructed; to block expression of the homologous *Salmonella* Typhimurium O-antigen, the *rfbN* gene, which encodes the rhamnosyl transferase that is responsible for the addition of rhamnose sugar to Und-PP-Gal of the O-antigen unit (Reeves et al., 2013), was deleted from SLT19, generating SLT20 (ATCC14028 Δ*asd* Δ*rfbN*); and to regulate the expression of the homologous O-antigen by exogenous arabinose provided during *in vitro* growth, the *rfbN* promoter was replaced with the *araC* P_BAD_ promoter, and the gene segment T *araC* P_BAD_ *rfbN* was inserted into the *pagL* site, generating SLT22 (ATCC14028 Δ*asd* Δ*rfbN* Δ*pagL*::T *araC* P_BAD_ *rfbN*). Then, pCZ11 or pCZb1 was introduced into the three Δ*asd* mutants, SLT19, SLT20, and SLT22, generating three recombinant strains, SLT19 (pCZ11), SLT20 (pCZ11), and SLT22 (pCZ11), and three control strains SLT19 (pCZb1), SLT20 (pCZb1), and SLT22 (pCZb1). The Asd^+^ plasmid and the Δ*asd* mutant constituted the balanced-lethal vector-host system for the stable expression of exogenous genes.

**Figure 1:**
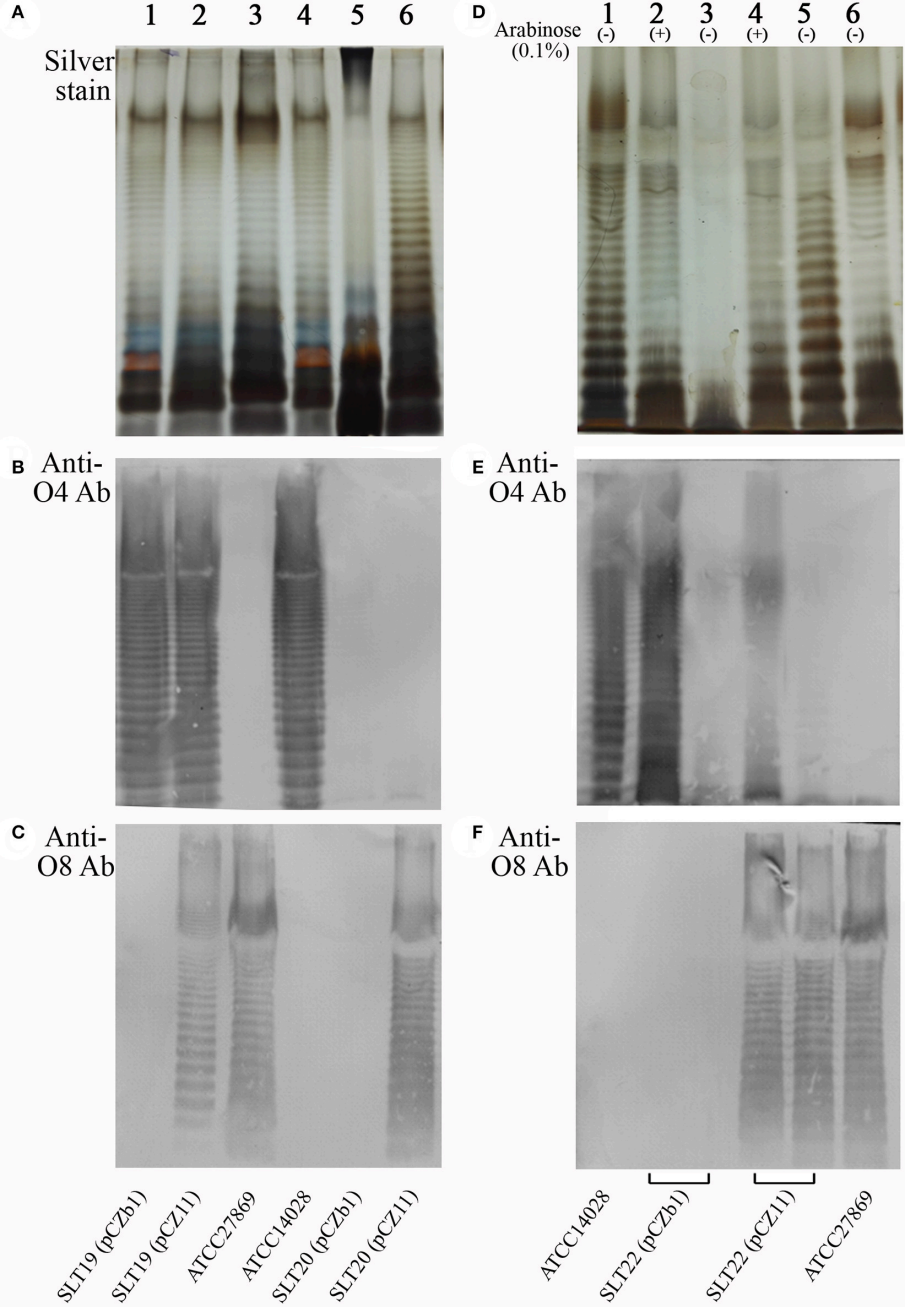
LPS analysis by silver staining and Western immunoblotting. **(A–C)** LPS extracted from SLT19 (pCZb1) (lane 1), SLT19 (pCZ11) (lane 2), ATCC27869 (lane 3), ATCC14028 (lane 4), SLT20 (pCZb1) (lane 5) and SLT20 (pCZ11) (lane 6) was subjected to SDS-PAGE followed by silver staining **(A)** and immunoblotting analysis using O:4-specific antiserum **(B)** and O:8-specific antiserum **(C)**. **(D–F)** LPS extracted from ATCC14028 (lane 1), SLT22 (pCZb1) grown with arabinose (lane 2) or without arabinose (lane 3), SLT22 (pCZ11) grown with arabinose (lane 4) or without arabinose (lane 5), and ATCC27869 (Lane 6) was subjected to SDS-PAGE followed by silver staining **(D)** and immunoblotting using O:4-specific antiserum **(E)** and O:8-specific antiserum **(F)**.

Silver staining and Western immunoblotting were used to analyze O-antigen expression. Silver staining showed that the wild-type *Salmonella* Newport ATCC27869 and *Salmonella* Typhimurium ATCC14028 produced typical LPS ladders (Figure 1A, lanes 3 and 4) representative of the smooth LPS phenotype. As expected, the SLT19 (pCZb1), SLT19 (pCZ11) and SLT20 (pCZ11) strains displayed smooth LPS phenotypes (Figure 1A, lanes 1, 2 and 6), whereas the control strain SLT20 (pCZb1) with an *rfbN* mutation displayed a rough LPS phenotype (Figure 1A, lane 5). Western immunoblotting demonstrated that SLT19 (pCZ11) expressed both the homologous and heterologous O-antigens (Figures 1B,C, lane 2). Conversely, the control strain SLT19 (pCZb1) only expressed the homologous O-antigen (Figures 1B,C, lane 1). The recombinant strain SLT20 (pCZ11) only expressed the heterologous O-antigen (Figures 1B,C, lane 6), while the control strain SLT20 (pCZb1) did not express either the homologous or heterologous O-antigen (Figures 1B,C, lane 5). Additionally, the recombinant strain SLT22 (pCZ11) expressed O-antigens in an arabinose-regulated manner: both types of O-antigens were expressed when the growth medium was supplemented with arabinose (Figures 1D–F, lane 4), but only the heterologous O-antigen was expressed in the absence of arabinose (Figures 1D–F, lane 5). In contrast, the control strain SLT22 (pCZb1) expressed the homologous O-antigen, but not the heterologous O-antigen, when grown with arabinose (Figures 1D–F, lane 2) and did not express either the homologous or heterologous O-antigen when grown without arabinose (Figures 1D–F, lane 3). Thus, SLT22 (pCZ11) co-expressed two types of O-antigens in the presence of arabinose, similar to SLT19 (pCZ11), and exclusively expressed the heterologous *Salmonella* Newport O-antigen when grown without arabinose, similar to SLT20.

### The effects of heterologous O-antigen expression on bacterial phenotypes and virulence

**Figure 2:**
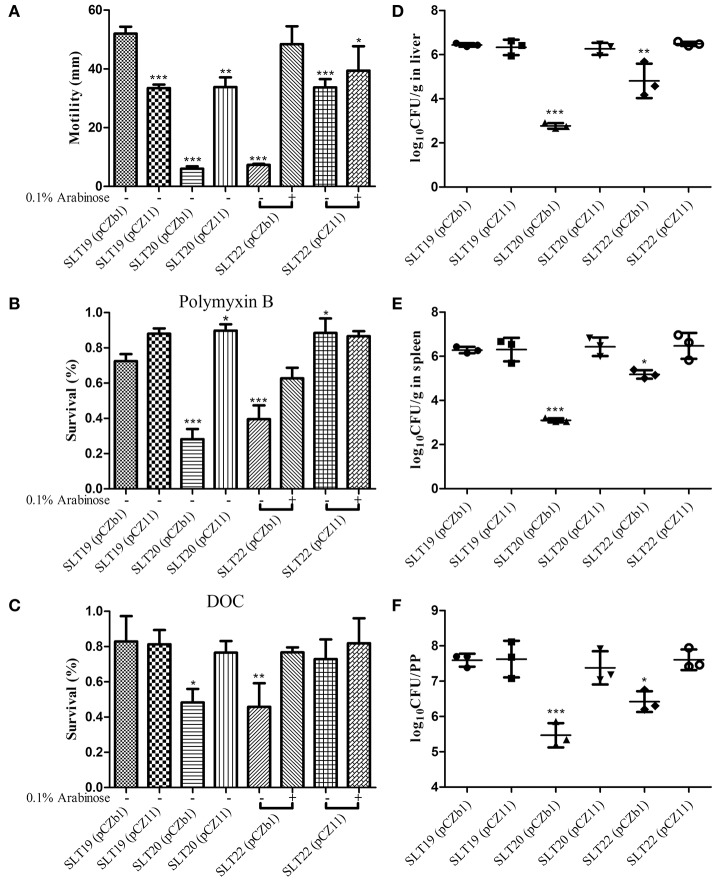
Biological activities and colonization of the *Salmonella* Typhimurium strains. **(A)** The swimming assay. SLT19 (pCZb1), SLT19 (pCZ11), SLT20 (pCZb1), SLT20 (pCZ11) and the two arabinose-regulated strains SLT22 (pCZb1) and SLT22 (pCZ11) were grown in LB broth with or without 0.1% arabinose. Then, 6 μl of the bacterial suspension (~1 × 10^8^ CFU/ml) was spotted onto the middle of LB plates containing 0.25% agar with or without arabinose, and the diameter of the colonies was measured after incubation at 37°C for 6 h. **(B,C)** The sensitivity assays. The six *Salmonella* Typhimurium strains were cultured as described in **(A)**, and 100 μl of each bacterial culture (~1 × 10^6^–5 × 10^6^ CFU) was inoculated with or without polymyxin B at a final concentration of 0.12 μg/ml or DOC at a final concentration of 4 mg/ml for 1 h at 37°C. The survival rate was calculated the following day. **(D–F)** Colonization. Groups of BALB/c mice (*n* = 4/group) were orally inoculated with 1 × 10^9^ CFU of each indicated strain. Viable bacteria were recovered from the liver **(D)**, spleen **(E)**, and PPs **(F)** 6 days after infection. The number of bacteria in each tissue was calculated as log_10_CFU/g. The asterisk above the error bar indicates significance compared with the SLT19 (pCZb1) group. ^*^p < 0.05, ^**^p < 0.01, ^***^p < 0.001.

As the O-antigen performs a key role in bacterial virulence, we evaluated whether the altered O-antigen profiles of the recombinant strains influenced the biological activities of *Salmonella* Typhimurium, including swimming, resistance to polymyxin B and DOC, colonization and virulence (LD_50_). Interestingly, compared with the parent strain SLT19 (pCZb1), SLT19 (pCZ11), SLT20 (pCZ11), and SLT22 (pCZ11) (grown with or without arabinose) exhibited decreased swimming ability, while SLT22 (pCZb1) (grown with arabinose), which had the homologous O-antigen profile, showed a similar swimming ability (Figure 2A). The control strains with the rough LPS phenotype, SLT20 (pCZb1) and SLT22 (pCZb1) (without arabinose), had very little swimming ability (Figure 2A). Compared with SLT19 (pCZb1), SLT20 (pCZ11), and SLT22 (pCZ11) (grown without arabinose), which displayed heterologous O-antigen profiles, showed slightly increased resistance to polymyxin B, but not DOC, while the strains SLT19 (pCZ11) and SLT22 (pCZ11) (grown with arabinose), which displayed chimeric O-antigen profiles, and SLT22 (pCZb1) (grown with arabinose), which displayed the homologous LPS profile, showed similar resistance to polymyxin B and DOC (Figures 2B,C). In addition, bacterial resistance to polymyxin B and DOC was dramatically reduced in SLT20 (pCZb1) and SLT22 (pCZb1) (without arabinose), which had rough LPS phenotypes, compared with that in SLT19 (pCZb1) (Figures 2B,C).

Regarding bacterial colonization in BALB/c mouse tissues, the recombinant strains SLT19 (pCZ11), SLT20 (pCZ11) and SLT22 (pCZ11) colonized the liver, spleen and PP at a high level that was similar to that of SLT19 (pCZb1), while the colonization level in all three tissues was significantly decreased for the SLT20 (pCZb1) and SLT22 (pCZb1) groups compared with the SLT19 group (pCZb1) (Figures 2D–F). Furthermore, the LD_50_ of SLT19 (pCZ11), SLT20 (pCZ11) and SLT22 (pCZ11) was 1.4 × 10^6^, 2.7 × 10^6^, and 8 × 10^5^ CFU, respectively, which was similar to that of the SLT19 (pCZb1) and wild-type strain ATCC14028 (LD_50_ of 5 × 10^5^ CFU). Conversely, the LD_50_of SLT20 (pCZb1) and SLT22 (pCZb1) declined substantially, to more than 10^9^ CFU (Table 2).

### Attenuation of the virulence of the recombinant strains

The *crp* gene, which encodes the global regulator CRP, which is associated with the utilization of carbon sources as well as bacterial virulence (Poncet et al., 2009), was deleted from SLT19 (pCZb1), SLT19 (pCZ11), SLT20 (pCZb1), SLT20 (pCZ11), SLT22 (pCZb1), and SLT22 (pCZ11) to attenuate the virulence, generating three recombinant vaccine strains harboring the plasmid pCZ11, SLT25 (SLT19 Δ*crp*), SLT26 (SLT20 Δ*crp*), and SLT27 (SLT22 Δ*crp*), and three control vaccine strains, SLT25 (pCZb1), SLT26 (pCZb1), and SLT27 (pCZb1). Western immunoblotting demonstrated that SLT25 (pCZ11), SLT26 (pCZ11), and SLT27 (pCZ11) displayed similar LPS phenotypes as SLT19 (pCZ11), SLT20 (pCZ11), and SLT22 (pCZ11) (Figure S2). SLT25 (pCZ11) (Figures S2A,B, lane 3), and SLT27 (pCZ11) (when grown with arabinose) (Figures S2C,D, lane 4) co-expressed both types of O-antigens; SLT26 (pCZ11) (Figures S2A,B, lane 5), and SLT27 (pCZ11) (when grown without arabinose) (Figures S2C,D, lane 5) expressed only the heterologous O-antigen. In contrast, the control vaccine strain SLT25 (pCZb1) (Figures S2A,B, lane 2) and SLT27 (pCZb1) (when grown with arabinose) (Figures S2C,D, lane 2) only expressed the homologous O-antigen; SLT26 (pCZb1) (Figures S2A,B, lane 4) and SLT27 (pCZb1) (when grown without arabinose) (Figures S2C,D, lane 3) showed rough LPS phenotypes. Furthermore, the LD_50_ for each of the six strains was determined in BALB/c mice. All six strains were highly attenuated, and their LD_50_ values were more than 10^9^ CFU, which was at least four orders of magnitude higher than that of the wild-type strain ATCC14028 (LD_50_ of 5 × 10^5^ CFU) (Table 2).

### Measurement of plasmid stability *in vitro* and *in vivo*

**Figure 3:**
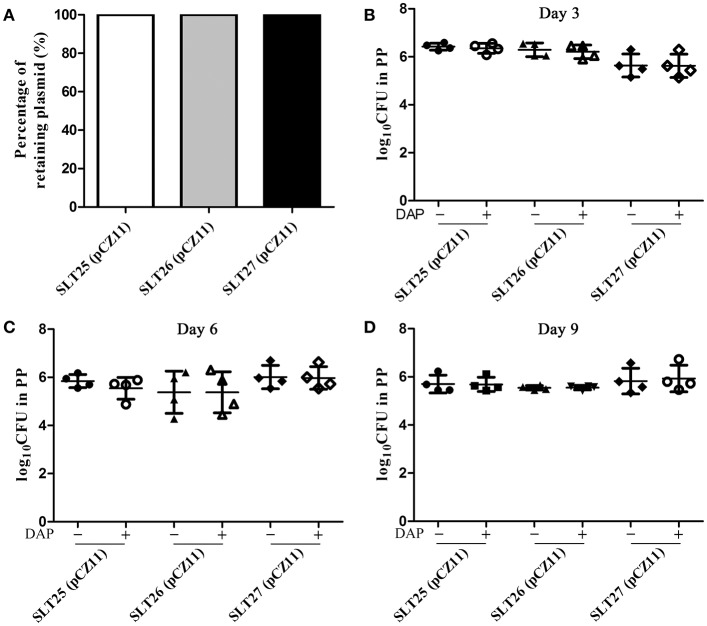
Stability assay of the Asd^+^ plasmid pCZ11 in the *Salmonella* vaccine strains. **(A)** Plasmid stability *in vitro*. The recombinant vaccine strains SLT25 (pCZ11), SLT26 (pCZ11), and SLT27 (pCZ11) were subjected to passage for 50 generations in LB containing DAP (non-selective medium). After the fifth passage, 100 single colonies were selected and streaked on LB agar plates without DAP (selective media) and on LB plates containing DAP (non-selective media). Plasmid stability was determined as the percentage of the 100 selected colonies that grew on the selective media. **(B–D)** Plasmid stability *in vivo*. Groups of BALB/c mice (12 mice/per group) were inoculated orally with ~1 × 10^9^ CFU of SLT25 (pCZ11), SLT26 (pCZ11), or SLT27 (pCZ11). PPs were collected from four mice of each group at 3 days **(B)**, 6 days **(C)**, and 9 days **(D)** post-infection and were weighed and homogenized in BSG. Then, appropriate dilutions were plated onto LB agar plates without DAP (selective media) and LB plates containing DAP (non-selective media) to determine the number of viable bacteria.

Stable maintenance of plasmid pCZ11 over some generations is an essential requirement to ensure the efficacy of recombinant vaccine strains. The stability of plasmid pCZ11 in the recombinant vaccine strains SLT25, SLT26 and SLT27 was determined *in vitro* by plating actively growing cultures on both selective and non-selective medium for 50 generations. As shown in Figure 3A, the percent retention of the Asd^+^ plasmid pCZ11 was 100% in SLT25, SLT26 and SLT27. To measure the stability *in vivo*, groups of BALB/c mice were orally inoculated with 10^9^ CFU of SLT25 (pCZ11), SLT26 (pCZ11) or SLT27 (pCZ11). The plasmid stability and persistence of the bacteria *in vivo* were investigated in the PPs at 3, 6, and 9 days post-infection. The recovered bacteria from the PPs were diluted and plated onto LB plates and LB plates with DAP. As shown in Figures 3B–D, the bacterial loads of all the vaccine strain-infected groups calculated from the LB plates supplemented with DAP were similar to those from the plates without DAP at 3, 6, and 9 days post-infection, indicating that the plasmid pCZ11 was stably retained in the recombinant vaccine strains SLT25, SLT26, and SLT27 *in vivo* for 9 days.

### Antibody responses and serum bactericidal effects after immunization with the generated vaccine strains

**Figure 4:**
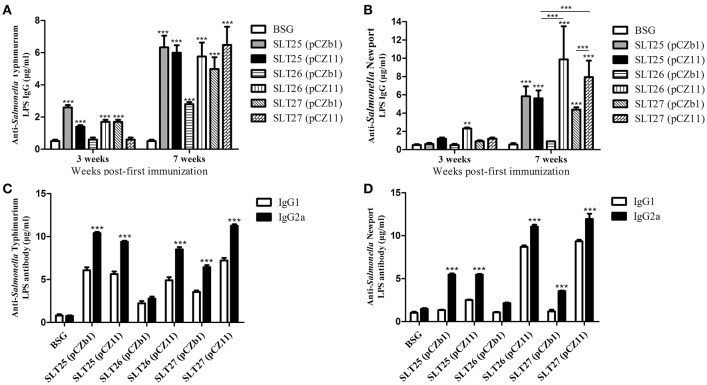
ELISA assay. **(A,B)** Serum total IgG responses. Groups of BALB/c mice were inoculated with SLT25 (pCZb1), SLT25 (pCZ11), SLT26 (pCZb1), SLT26 (pCZ11), SLT27 (pCZb1), SLT27 (pCZ11), or BSG twice at an interval of 4 weeks. Serum samples (*n* = 8/group) were collected at 3 weeks and 7 weeks post-first immunization. Serum IgG specific to *Salmonella* Typhimurium LPS **(A)** and *Salmonella* Newport LPS **(B)** was detected by quantitative ELISA in each group. **(C,D)** Serum IgG2a and IgG1 responses. Serum IgG2a and IgG1 specific to *Salmonella* Typhimurium LPS **(C)** and *Salmonella* Newport LPS **(D)** was detected by quantitative ELISA in each group at 7 weeks post-first immunization. The asterisk above the error bar indicates significance compared with the BSG control group. The asterisk above the line indicates significance between the two indicated groups. ^**^p < 0.01, ^***^p < 0.001.

To evaluate the immunogenicity of the delivered O-antigens, BALB/c mice were immunized orally with ~10^9^ CFU of each of the six vaccine strains or BSG twice at an interval of 4 weeks. ELISA results showed that compared with the control BSG group, a weak but significant serum total IgG response specific to the homologous LPS was induced in the SLT25 (pCZb1), SLT25 (pCZ11), SLT26 (pCZ11), and SLT27 (pCZb1) groups, but not the SLT26 (pCZb1) and SLT27 (pCZ11) groups, 3 weeks post-first immunization (Figure 4A). An increased total IgG response to the homologous LPS was observed in all of the vaccine groups 7 weeks post-first immunization; however, the IgG amount was much lower in the SLT26 (pCZb1) group than in the other five groups post-second immunization (*p* < 0.001, Figure 4A). For the heterologous LPS-specific serum IgG responses, only the vaccine strain SLT26 (pCZ11) produced significantly higher IgG levels than the BSG group 3 weeks post-first immunization, and all vaccine-immunized groups except the SLT26 (pCZb1) group produced increased IgG responses 7 weeks post-first immunization (Figure 4B). Immunization with SLT25 (pCZ11) produced a serum IgG level similar to that induced by the control SLT25 (pCZb1) strain, while the SLT27 (pCZ11) group exhibited a higher IgG level than the control SLT27 (pCZb1) group post-second immunization. Additionally, the antibody amounts detected in the SLT26 (pCZ11) and SLT27 (pCZ11) groups were higher than that detected in the SLT25 (pCZ11) group post-second immunization (Figure 4B). Furthermore, the antibody levels of serum IgG subtypes IgG2a, which is indicative of a Th1-type immune response, and IgG1, which is indicative of a Th2-type immune response, were also determined post-second immunization. Immunization with all six vaccines elicited significantly higher levels of IgG2a than IgG1 in response to the homologous O-antigens (Figure 4C). Interestingly, compared to the antibody levels in the BSG group, no significant IgG1 responses against the heterologous O-antigen were induced in the three control vaccine groups, SLT25 (pCZb1), SLT26 (pCZb1) and SLT27 (pCZb1), whereas significantly higher IgG2a levels against the heterologous O-antigen were produced in all of the vaccine-immunized groups except for the SLT26 (pCZb1) group (*p* < 0.001, Figure 4D). The IgG2a levels were significantly higher than the IgG1 levels in all vaccine-immunized groups except the SLT26 (pCZb1) group (Figure 4D). These results suggested that the recombinant vaccines stimulated predominantly Th1-type immune responses post-second immunization.

**Figure 5:**
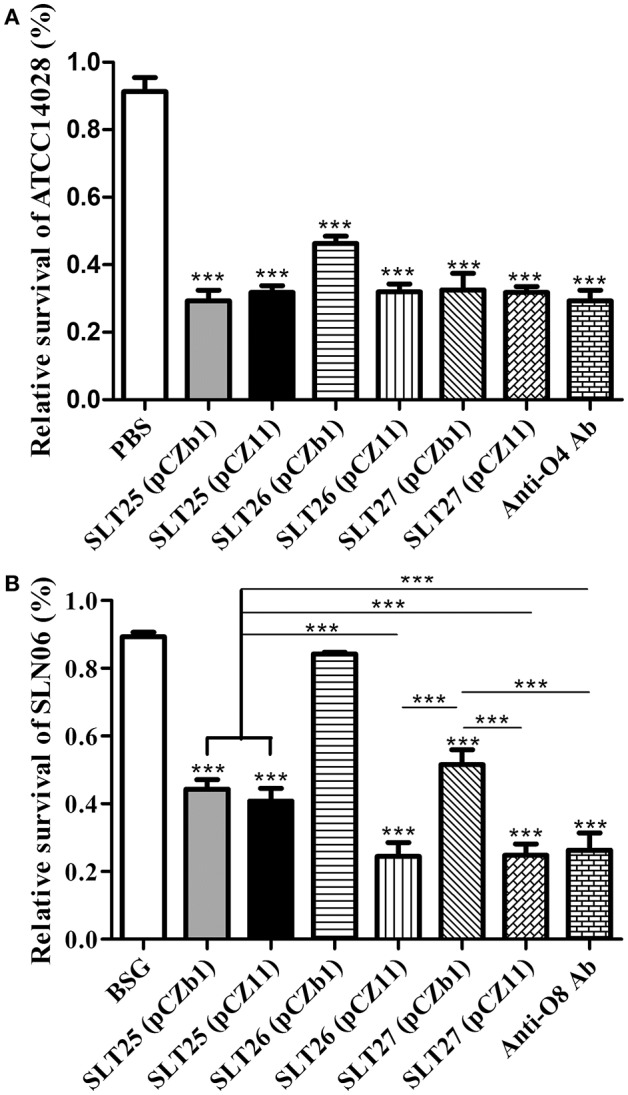
Serum bactericidal assay. **(A,B)** *Salmonella* Typhimurium ATCC14028 **(A)** or *Salmonella* Newport SLN06 **(B)** was incubated with heat-inactivated serum collected at week 7 post-first immunization from mice in each immunized group. Active guinea pig complement was added to or omitted from the mixture. The relative survival was measured at 1.5 h post-incubation. The asterisk above the error bar indicates significance compared with the BSG control group. The asterisk above the line indicates significance between the two indicated groups. ^***^p < 0.001.

The serum collected from all immunized groups at 7 weeks post-first immunization were used to measure bactericidal activity against the homologous *Salmonella* Typhimurium strain ATCC14028 or the heterologous *Salmonella* Newport strain SLN06. The bacteria were incubated with 25% heat-inactivated serum plus active guinea pig complement or no complement for 1.5 h, and the relative survival was then calculated as the percent CFU in each serum with active complement compared to the CFU of the same serum with no complement. The sera of all vaccine-immunized groups elicited significant bactericidal effects against ATCC14028 compared with the BSG group (Figure 5A). The bacterial survival rates in all of the vaccine-immunized groups were much lower than that of the BSG group and were similar to that of the positive O:4-specific antiserum, and the survival rate of the SLT26 (pCZb1) group was significantly higher than that of the other five vaccine groups (*p* < 0.001, Figure 5A). Furthermore, the sera from all vaccine-immunized groups except for the SLT26 (pCZb1) group had significant bactericidal effects against SLN06 compared with the BSG group; however, the bactericidal levels in these vaccine groups were quite diverse. The sera from mice vaccinated with the two recombinant vaccine strains SLT26 (pCZ11) and SLT27 (pCZ11) were able to kill SLN06 at a similar level, which was comparable to that of the positive O:8-specific antiserum but much better than those of the remaining vaccine strains, SLT25 (pCZb1), SLT25 (pCZ11) and SLT27 (pCZb1) (Figure 5B).

### Protection efficacies provided by the vaccine strains

**Figure 6:**
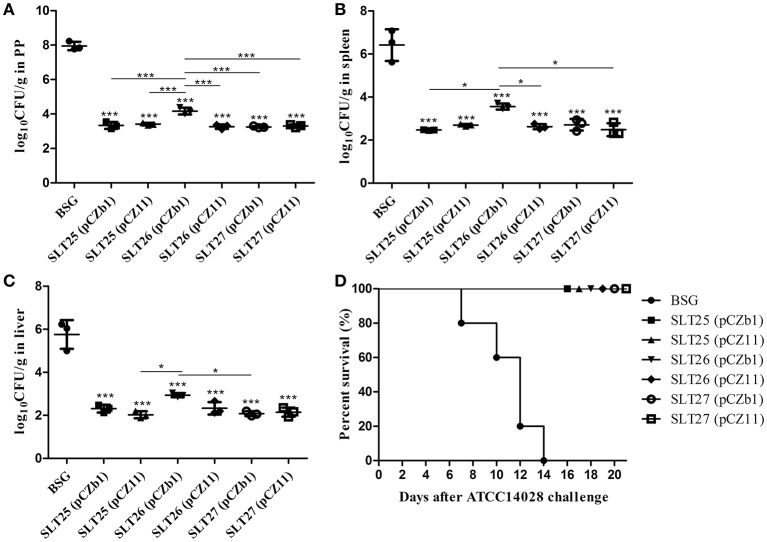
Bacterial loads and survival of mice post-challenge with ATCC14028. The BALB/c mice in each immunization group were orally inoculated with a lethal dose of *Salmonella* Typhimurium ATCC14028. Six days after oral inoculation, the PPs **(A)**, spleen **(B)**, and liver **(C)** were collected from mice in each group (*n* = 4/group). Then, the bacteria in the tissues were recovered and measured as log_10_CFU/g. Animal mortality (*n* = 12/group) was recorded daily for 21 days post-challenge **(D)**. The asterisk above the error bar indicates significance compared with the BSG control group. The asterisk above the line indicates significance between the two indicated groups. ^*^p < 0.05, ^***^p < 0.001.

The mice in each immunized group were challenged by oral inoculation with at least 100 × LD_50_ of the *Salmonella* Typhimurium virulent strain ATCC14028 or *Salmonella* Newport virulent strain SLN06 at 1 month post-second immunization. The bacterial loads in the mouse tissues were measured at 6 days post-challenge. After the challenge with ATCC14028, the PPs, spleen and liver from all vaccine-immunized groups had significantly fewer bacterial CFU compared with the BSG group (Figures 6A–C). The bacterial count of the SLT26 (pCZb1)-immunized group was higher than that of the other five vaccine groups in PP (Figure 6A), the SLT25 (pCZb1) and SLT27 (pCZ11) groups in the spleen (Figure 6B), and the SLT25 (pCZ11) and SLT27 (pCZb1) groups in the liver (Figure 6C). Furthermore, no mortality was observed in any of the vaccine-immunized groups, while all 12 mice in the BSG group succumbed to the challenge within 14 days (Figure 6D).

**Figure 7:**
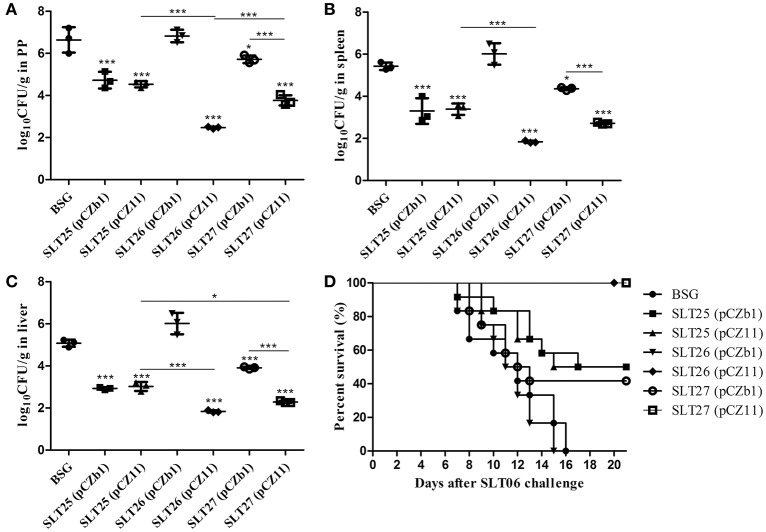
Bacterial load and survival of mice post-challenge with SLN06. The BALB/c mice in each immunization group were orally inoculated with a lethal dose of *Salmonella* Newport SLN06. Six days after oral inoculation, the PPs **(A)**, spleen **(B)** and liver **(C)** were collected from mice in each group (*n* = 4/group). Then, the bacteria in the tissues were recovered and measured as log_10_CFU/g. Animal mortality (*n* = 12/group) was recorded daily for 21 days post-challenge **(D)**. The asterisk above the error bar indicates significance compared with the BSG control group. The asterisk above the line indicates significance between the two indicated groups. ^*^p < 0.05, ^***^p < 0.001.

After the SLN06 challenge, the mice immunized with SLT25 (pCZb1), SLT25 (pCZ11), SLT26 (pCZ11), SLT27 (pCZb1), and SLT27 (pCZ11), but not SLT26 (pCZb1), showed a significant reduction in the number of CFU of *Salmonella* Newport in the PPs, spleen and liver compared with the BSG-immunized mice. The CFU numbers in these three tissues of mice immunized with SLT25 (pCZ11) were nearly the same as those observed in the SLT25 (pCZb1)-immunized mice, while the CFU numbers in all three tissues were also significantly reduced in mice immunized with SLT27 (pCZ11) compared with the SLT27 (pCZb1)-dosed group (Figures 7A–C). In the PP, the number of CFU was lower in the SLT26 (pCZ11) group than in the SLT25 (pCZ11) and SLT27 (pCZ11) groups (Figure 7A). In the spleen and liver, the number of CFU in the SLT26 (pCZ11) group was similar to that in the SLT27 (pCZ11) group and lower than that observed in the SLT25 (pCZ11) group (Figures 7B,C). Additionally, a significant reduction in the number of CFU in the liver was observed in the group of mice immunized with SLT27 (pCZ11) compared with the group immunized with SLT25 (pCZ11) (Figure 7C). Furthermore, all 12 mice in the SLT26 (pCZ11)- and SLT27 (pCZ11)-immunized groups survived the *Salmonella* Newport SLN06 challenge, suggesting 100% protection. Immunization with SLT25 (pCZb1) or SLT25 (pCZ11) resulted in 50% survival, and immunization with SLT27 (pCZb1) resulted in 41.7% survival. By contrast, all of the mice in the SLT26 (pCZb1) and BSG groups succumbed to the challenge (Figure 7D).

**Figure 8:**
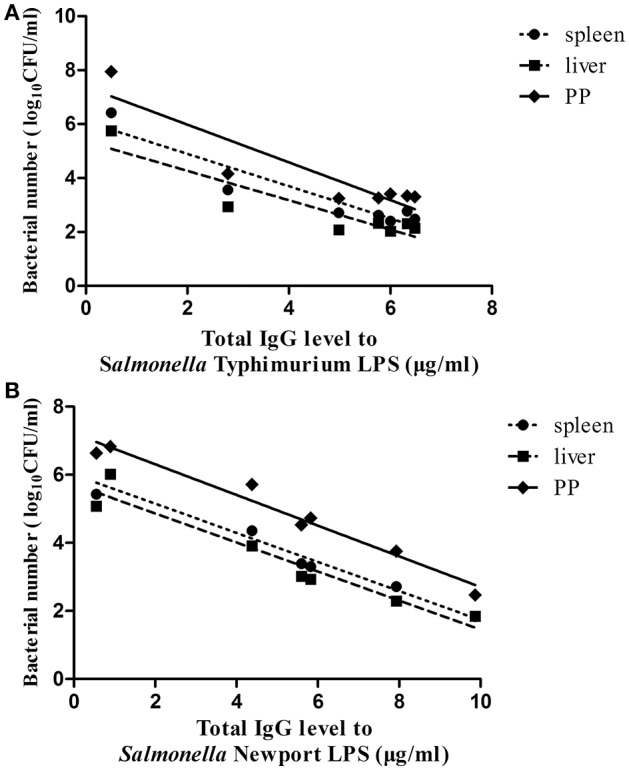
The correlation between serum total IgG levels post-second immunization and bacterial loads post-challenge. **(A,B)** Spearman's rank correlation analysis was applied to determine the correlations between the total IgG levels against *Salmonella* Typhimurium LPS post-second immunization and the bacterial loads in the PP, spleen and liver in all of the immunized groups post-ATCC14028 challenge **(A)**, as well as the correlations between the total IgG levels against *Salmonella* Newport LPS post-second immunization and the bacterial loads in the PP, spleen and liver in all of the immunized groups post-SLN06 challenge **(B)**. Lines represent the non-linear regression of the data, and each closed circle is the value of one of the seven immunized groups.

Finally, to evaluate the role of the O-antigen-mediated antibody response in protection against lethal *Salmonella* Typhimurium or *Salmonella* Newport challenge, the correlation between the mean value of the total IgG levels against *Salmonella* Typhimurium LPS or *Salmonella* Newport LPS in each immunized group post-second immunization and the mean value of the bacterial loads of each immunized group in the PP, spleen and liver post-ATCC14028 or SLN06 challenge was analyzed using Spearman's rank correlation test. The results showed that the IgG levels against *Salmonella* Typhimurium LPS showed a negative but not statistically significant correlation with the bacterial load post-ATCC14028 challenge in the PP, spleen and liver (Figure 8A), with r values of −0.6429, −0.679, and −0.3571, respectively. In contrast, the IgG levels against *Salmonella* Newport LPS were significantly and negatively correlated to the bacterial load post-SLN06 challenge in the PP (Figure 8B, Spearman r = −0.9286, *p* = 0.0067), spleen (Figure 8B, *r* = −0.9643, *p* = 0.0028) and liver (Figure 8B, *r* = −0.9643, *p* = 0.0028), indicating that heterologous O-antigen-specific serum IgG acted as a functional correlate of protection against *Salmonella* Newport challenge.

## Discussion

NTS serovars are important causes of gastrointestinal disease in healthy people and of invasive disease in immunocompromised individuals worldwide (Majowicz et al., 2010; Feasey et al., 2012). Vaccination is one of the best forms of prophylaxis against the development of infectious disease. An effective *Salmonella* vaccine with broad serovar coverage is urgently needed to control infections by a variety of NTS serovars. In this study, we developed bivalent *Salmonella* vaccines through the recombinant expression of the heterologous *Salmonella* Newport O-antigen in *Salmonella* Typhimurium. This strategy was applied for four reasons: (1) O-antigen-specific antibodies can mediate robust protection against *Salmonella* Typhimurium, *Salmonella* Enteritidis or *Salmonella* Paratyphi infections (MacLennan et al., 2014), although this protective role has not been observed in *Salmonella* Newport infections; (2) O-antigen polymorphism is a mechanism used to evade cross-immunity between different *Salmonella* serovars, such as Enteritidis and Typhimurium (Hormaeche et al., 1996; Kingsley and Baumler, 2000); (3) *Salmonella* Typhimurium is an ideal vaccine vector for the delivery of exogenous antigens, including O-antigen, to the host immune system (Bridge et al., 2016), and a set of recombinant vaccine technologies has been developed for this serovar (Wang et al., 2013); and (4) a potential advantage of O-antigen-based vaccines is cross-protection against other serovars within the same serogroup because all serovars in one serogroup express the same dominant O-antigen epitopes, which has been demonstrated in the serogroup D serovars *Salmonella* Enteritidis and *Salmonella* Gallinarum (Chacana and Terzolo, 2006). The Asd-based balanced-lethal vector-host system was utilized to construct three bivalent *Salmonella* vaccines. We proved that the recombinant Asd^+^ plasmid pCZ11, which expressed the heterologous O-antigen, was stably retained in the three recombinant *Salmonella* Typhimurium vaccines *in vitro* and *in vivo*, consistent with previous reports utilizing the balanced-lethal vector-host system (Xin et al., 2012). The duration time examined *in vivo* for the recombinant strains carrying pCZ11 was at least 9 days, which is sufficient to stimulate protective immunity to *Salmonella* infection, as a previous report has demonstrated (Griffin and McSorley, 2011). Our study demonstrates that immunization with the constructed bivalent vaccine strains SLT26 (pCZ11), which has the heterologous O-antigen phenotype, and SLT27 (pCZ11), which has an arabinose-dependent O-antigen phenotype, stimulate strong serum IgG responses, including IgG2a and IgG1, against two types of O-antigen and provide full protection against lethal challenges with the homologous and heterologous *Salmonella* strains.

LPS O-antigen forms the outer layer of *Salmonella* and other gram-negative bacteria, protecting the bacteria from extreme environments, antimicrobial peptides and other host defense factors. We found that SLT20 (pCZb1) and SLT22 (pCZb1) (grown without arabinose), which displayed rough LPS phenotypes due to the *rfbN* mutation, were significantly defective in swimming, resistance to polymyxin B and DOC, colonization and virulence in mice, similar to findings in previous reports of a *Salmonella* Typhimurium Δ*rfbP* mutant (Kong et al., 2011) and a *Salmonella* Enteritidis strain with transposon insertions in the *rfbN* gene (Addwebi et al., 2014), confirming the essential role of O-antigen in bacterial virulence, motility, and immune evasion. Interestingly, we also found that the exclusive expression of the *Salmonella* Newport O-antigen in *Salmonella* Typhimurium decreases bacterial swimming. Flagellum-mediated motility is considered a basic function of *Salmonella* that contributes to its interactions with host cells during pathogenesis; however, the mechanism underlying the function of O-antigen in motility has not been fully elucidated. An early study suggested that the loss of surface O-antigen inhibits the swarming motility of *Salmonella* Typhimurium because it generally acts to increase the surface “wettability” required for bacterial colony expansion (Toguchi et al., 2000). Recent reports demonstrated that auto-aggregation and a defect in flagellar expression were responsible for the reduced swimming motility of a *Salmonella* Choleraesuis rough strain with a *wzx* gene deletion (Zhou et al., 2014), and the inactivation of genes involved in LPS synthesis decreases flagellar protein production in *Salmonella* Typhimurium (Liu et al., 2016). Thus, whether a similar mechanism for defective swimming motility occurs in the *Salmonella* Δ*rfbN* mutant and whether expression of the *Salmonella* Newport O-antigen has an adverse effect on flagellar expression still need to be addressed. Additionally, we demonstrated that the exclusive expression of the *Salmonella* Newport O-antigen in *Salmonella* Typhimurium has no influence on bacterial colonization and virulence in mice, indicating that O-antigen expression itself, rather than O-antigen variety, was vital for *Salmonella* virulence. An analogous observation was also found in a previous report in which the initial interaction between newly hatched chickens and *Salmonella* was found to be dependent on the presence of O-antigen but not serovar classification (Varmuzova et al., 2014).

As an ideal vaccine vector, live attenuated *Salmonella* vaccines have been utilized to deliver O-antigens from other pathogenic bacteria, such as *Shigella sonnei* (Dharmasena et al., 2013) and *Burkholderia mallei* (Moustafa et al., 2015), exhibiting protection against lethal challenge. However, a *Salmonella* vaccine strain expressing *Pseudomonas aeruginosa* O-antigen exhibits only partial protection against lethal challenge (Bridge et al., 2016). All of these developed *Salmonella* vectors possess complete homologous LPS phenotypes. The co-expression of two types of O-antigens may attenuate the immunogenicity of the heterologous O-antigen and the resulting protection potency. Therefore, to avoid this potentially negative effect in our study, in addition to the construction of SLT25 (pCZb1), which co-expressed two types of O-antigen, we also constructed two other recombinant vaccine strains, SLT26 (pCZ11), which expressed only the heterologous O-antigen, and SLT27 (pCZ11), which expressed two types of O-antigen when exogenous arabinose was supplied during *in vitro* growth and expressed only the heterologous O-antigen *in vivo*, as the expression of the homologous O-antigen ceases after *Salmonella* invasion due to cell division and the absence of arabinose in the gut-associated lymphoid tissues (Kong et al., 2008). Not unexpectedly, SLT26 (pCZ11) and SLT27 (pCZ11) immunization induced higher levels of serum IgG specific to the heterologous O-antigen and better bactericidal effects against *Salmonella* Newport than immunization with SLT25 (pCZ11). Correspondingly, in contrast to the 50% heterologous protection provided by SLT25 (pCZ11) immunization, the other two vaccines conferred full protection against *Salmonella* Newport infection. We speculate that the presence of the homologous O-antigen influenced the epitope exposure of the heterologous O-antigen to B-cells or decreased the amount of heterologous O-antigen expressed in SLT25 (pCZ11), resulting in its poorer immunogenicity. The results suggest that the exclusive expression of a heterologous O-antigen might be a better strategy for developing an O-antigen-based bivalent *Salmonella* vaccine than the co-expression of two types of O-antigen.

However, the serum IgG levels induced against the homologous LPS and their related bactericidal effects to *Salmonella* Typhimurium post-immunization as well as the resultant bacterial clearance levels and protection efficacies after homologous challenge were similar between the three recombinant vaccine-immunized groups. In combination with the finding that immunization with SLT26 (pCZb1), which did not express the homologous LPS, also provided full homologous protection, the results suggest that the protective role of antibodies specific to the homologous O-antigen are not indispensable for the live attenuated *Salmonella* vaccines. This finding is in line with previous reports based on attenuated *Salmonella* Typhimurium rough strains with mutations in O-antigen synthesis genes (Nagy et al., 2006) and is similar to the role of Vi-mediated antibodies in the protection against *Salmonella* Typhi infections, as demonstrated by the protection conferred by the commercial Vi CPS and live Ty21a vaccines without the Vi antigen (MacLennan et al., 2014). Nevertheless, the presence of O-antigen may be essential for the efficacy of inactivated *Salmonella* vaccines, as the sera induced by inactivated rough strain immunization were not able to kill *Salmonella* (Rondini et al., 2013). Immunization with live *Salmonella* vaccines elicits robust humoral and T cell-mediated immune responses, both of which are required for preventing *Salmonella* infection (Wahid et al., 2015; Zhu et al., 2017). In addition to the protective antigen O-polysaccharide, specific immune responses stimulated by other *Salmonella* antigens, such as flagellin proteins and OMPs are strongly associated with protection against *Salmonella* infection (Mastroeni and Menager, 2003; Bergman et al., 2005). Thus, these kinds of immune responses are likely responsible for the full protection conferred by the vaccine strain SLT26 (pCZb1), which had a rough LPS phenotype, or SLT26 (pCZ11), which had a heterologous O-antigen profile. The finding that the serum IgG response against homologous LPS was negatively but not significantly correlated to the bacterial loads in tissues after homologous challenge might be attributable to the same cause.

Bacterial-specific Th1 immune responses including antibodies, CD4 T cells, cytokines are essential for the clearance of disseminated *Salmonella* infections, and Th1-promoting vaccines are likely to help prevent these infections (Ravindran and McSorley, 2005). All three of the recombinant vaccine strains stimulated more serum IgG2a (characteristic of a Th1-type response) than IgG1 (characteristic of a Th2-type response) against the heterologous LPS post-second immunization, indicating a biased Th1-type immune response. This finding was consistent with a number of studies in which recombinant antigens expressed in oral, attenuated *Salmonella* vaccines induced a predominately Th1 response, as did the vector itself (VanCott et al., 1996; Pathangey et al., 2009; Moustafa et al., 2015).

Interestingly, we also found that the SLT26 (pCZ11) strain, which expressed only the heterologous O-antigen, and the SLT25 (pCZb1) and SLT27 (pCZb1) strains, which expressed only the homologous O-antigen, stimulated specific serum IgG responses to both the homologous and heterologous LPS post-second immunization, indicating that there is cross-reactivity between the antibodies specific to *Salmonella* Typhimurium LPS and *Salmonella* Newport LPS. In other words, there are epitopes common to both *Salmonella* Typhimurium LPS and *Salmonella* Newport LPS. A similar observation was also demonstrated in a previous study in which monoclonal antibody against O:5 antigen expressed by *Salmonella* strains from group B (O-antigenic formula, 1,4,5,12) cross-reacted with an unidentified lipopolysaccharide epitope of the *Salmonella* serogroup C2 (O-antigenic formula, 6,8). The cross-reactivity between antibodies against two types of LPS also resulted in cross-protection as evidenced by the finding that immunization with SLT25 (pCZb1) or SLT27 (pCZb1) expressing only the homologous O-antigen resulted in a significant reduction in the bacterial loads in mouse tissues and provided 50 or 41.7% heterologous protection efficacy upon heterologous *Salmonella* Newport challenge, respectively; conversely, the mice in the rough SLT26 (pCZb1)-immunized group had bacterial loads similar to those in the mice in the BSG group, and all of these mice succumbed to the heterologous challenge. A previous report showed that a *Salmonella* Typhimurium Δ*crp* Δ*cya* mutant conferred significant but marginal protection against a challenge with group C2 serovars in chickens (Hassan and Curtiss, 1994). Our study suggests that the cross-reactivity of IgG against the LPS of Group B and Group C2 *Salmonella* might be partially responsible for the observed cross-protection. Finally, we also demonstrated that the amounts of serum total IgG against *Salmonella* Newport LPS were positively and significantly correlated to bacterial clearance after heterologous protection, proving the protective role of the *Salmonella* Newport O-antigen-mediated IgG response in the prevention of *Salmonella* Newport infection, similar to the essential role of O-antigens from extensively studied serovars such as *Salmonella* Typhimurium, *Salmonella* Enteritidis, and *Salmonella* Paratyphi (MacLennan et al., 2014). Therefore, the level of antibody response could become a marker for evaluating the protection efficacy of O-antigen-based vaccines against *Salmonella* infections.

In summary, we constructed three bivalent *Salmonella* vaccine strains, SLT25 (pCZ11), SLT26 (pCZ11), and SLT27 (pCZ11), with different LPS profiles by stable recombinant expression of the heterologous *Salmonella* Newport O-antigen in attenuated *Salmonella* Typhimurium. Expression of the heterologous O-antigen had no adverse effects on *Salmonella* Typhimurium colonization and virulence in mice but decreased bacterial swimming and susceptibility to polymyxin B. All three of the vaccine strains provided full protection against a *Salmonella* Typhimurium challenge. In contrast to the moderate heterologous protection provided by SLT25 (pCZ11), immunization with SLT26 (pCZ11) and SLT27 (pCZ11) conferred full protection against a *Salmonella* Newport challenge. Thus, the O-antigen-stimulated antibody response plays a protective role in preventing *Salmonella* Newport infection, and the expression of heterologous O-antigens in attenuated *Salmonella* Typhimurium strains is a promising strategy for the development of effective *Salmonella* vaccines with broad serovar coverage.

## Author contributions

XZ, QD, and AC designed the experiments, analyzed the data, and drafted the manuscript. XZ, QD, RJ, DZ, ML, MW, and SC performed the research. KS, QY, and YW participated in the animal experiments. XZ and AC edited the manuscript.

### Conflict of interest statement

The authors declare that the research was conducted in the absence of any commercial or financial relationships that could be construed as a potential conflict of interest.
