# Supplementary material for: Two Novel Salmonella Bivalent Vaccines Confer Dual Protection against Two Salmonella Serovars in Mice
Source: Front Cell Infect Microbiol. 2017 Sep 4;7:391. doi: 10.3389/fcimb.2017.00391 (PMC5591321; doi:10.3389/fcimb.2017.00391)
Supplement: Supplementary file 1 [file DataSheet1.docx]

***Supplementary Material:***

**Two Novel *Salmonella* Bivalent Vaccines Confer Dual Protection Against** **Two *Salmonella* Serovars In Mice**

Xinxin Zhao^*^, Qinlong Dai, Renyong Jia, Dekang Zhu, Mafeng Liu, Mingshu Wang, Shun Chen, Kunfeng Sun, Qiao Yang, Ying Wu, Anchun Cheng^*^

^*^**Correspondence:** Xinxin Zhao, xxinzhao@sicau.edu.cn; Anchun Cheng, [chenganchun@vip.163.com](mailto:chenganchun@vip.163.com).

**Table S1. Primers used in this study.**

| Primer | Sequence 5’-3’ |
| --- | --- |
| pSC101-2F | aggatcttcctcgaggactcctgttgatagatccag |
| pSC101ori-R | cgattgtctagacgaactgaatgtcacgaaaaag |
| Pasd-F | gttcgtctagacaatcgcccaacgacattttgcc |
| pSC101-2R | ttaattgcgttgcgcttccgaattcaacatcaggtagtg |
| pSC101-1F | atgttgaattcggaagcgcaacgcaattaatgtg |
| pSC101-1R | caacaggagtcctcgaggaagatcctttgatcttttctac |
| T1T2-F | gtacccggggatcctctgcagATaagcttggctgttttggcggatg |
| Ptrc-R | gaggatccccgggtaccgcggccgctctgtttcctgtgtgaaattg |
| C2 O-antigen-F | CACACAGGAAACAGAATGAATCGTATTATTAGAATGTTAGGTGTAG |
| C2 O-antigen-R | TCCGCCAAAACAGCCCTGTTTCGGATGCTCTTCACG |
| pCZb1-C2F | GAGCATCCGAAACAGGGCTGTTTTGGCGGATG |
| pCZb1-C2R | AATAATACGATTCATTCTGTTTCCTGTGTGAAATTGTTATC |
| D*rfbN*-1F | CGGGGTACCGTATGTGGTCGTACTGAC |
| D*rfbN*-1R | TTTGACGAAGTTATTTTGATTCCGCCAACC |
| D*rfbN*-2F | ATCAAAATAACTTCGTCAAAAGAGATAAAATAAATG |
| D*rfbN*-2R | TCCCCCCGGGGCATGTCTGACAGCTTTC |
| *rfbN*-F | CCCGGATCTTACAGATGAAAATAACATTAATTATTCCCAC |
| *rfbN*-R | GCGGCCGCATCCTGCAGGTTATTTTATCTCTTTTGACG |
| T*araC*P_BAD_*-*F | AAGGAAAAAAGCGGCCGCATTTCAGATAAAAAAAATCCTTAGC |
| T*araC*P_BAD_*-*R | TAATGTTATTTTCATCTGTAAGATCCGGGTATGG |
| D*asd*-1F | CGGGGTACCCGGCGCGATTGTCGGGATG |
| D*asd*-1R | CGCCCCATAAAGCGTTTTTTTCCTGCAAAG |
| D*asd*-2F | GGAAAAAAACGCTTTATGGGGCGCCGC |
| D*asd*-2R | TCCCCCCGGGGTCCGGCTTGGGTCTGGTGC |
| D*pagL*-1F | CGGGGTACCTGCGGATGAAGCTGCCGACC |
| D*pagL*-1R | CCTGCAGGATGCGGCCGCTGAAGTTGAATAACAATTAGCG |
| D*pagL*-2F | GCGGCCGCATCCTGCAGGCTCCACCACCATTTCAATGTC |
| D*pagL*-2R | TCCCCCCGGGAGACTATCTTTACTGGCAGG |
| D*crp*-1F | CGGGGTACCCGCAGTTGGTGACATTCTGACG |
| D*crp*-1R | TCTGACGGAAGCGCGGTTATCCTCTG |
| D*crp*-2F | ATAACCGCGCTTCCGTCAGAATGGCGC |
| D*crp*-2R | TCCCCCCGGGGCCATAGCCAGAACCAAAACCA |

**Figures**





**Figure S1. Schematic representation of plasmid construction.** (A) Construction of pCZb0. The DNA fragment containing the lambda t0 terminator (t0 T) and pSC101 origin from the plasmid pKS011 and *asd* gene cassette from ATCC14028 genome were joined by overlap PCR. The product was then assembled with the DNA fragment containing the TIT2 terminator, Ptrc promoter, *sacB* gene cassette and ampicillin-resistance cassette (AmpR) cloned from the plasmid pTRC-LIC to form the plasmid pCZb0 using the Gibson assembly method. (B) Construction of pCZb1. The control plasmid pCZb1 was generated by removal of *sacB* gene cassette from the pCZb0. (C) Construction of pCZ11. The gene segment including *wzx*-*wbaR*-*wbaL*-*wbaQ*-*wzy*-*wbaW*-*wbaZ* from the ATCC27869 genome was assembled with the entire coding sequence of plasmid pCZb1 by the Gibson assembly, generating the recombinant plasmid pCZ11.





**Figure S2. LPS analysis by Western immunoblotting.** (A and B) LPS extracted from *Salmonella* strains were subjected to SDS-PAGE followed by immunoblotting analysis using O:4-specific antiserum (A) and O:8-specific antiserum. Lane 1, *Salmonella* Typhimurium wild-type strain ATCC14028; lane 2, SLT25 (pCZb1); lane 3, SLT25 (pCZ11); lane 4, SLT26 (pCZb1); lane 5, SLT26 (pCZ11); lane 6, *Salmonella* Newport wild-type strain ATCC27869. (C and D) LPS extracted from ATCC14028 (lane 1), SLT27 (pCZb1) grown with arabinose (lane 2) or without arabinose (lane 3), SLT27 (pCZ11) grown with arabinose (lane 4) or without arabinose (lane 5), and ATCC27869 (lane 6) were subjected to SDS-PAGE followed immunoblotting using O:4-specific antiserum (C) and O:8-specific antiserum (D).
